# A Patch-Based Attention Framework for Automated Diabetic Retinopathy Severity Grading Using Fundus Images

**DOI:** 10.3390/diagnostics16213601

**Published:** 2026-11-01

**Authors:** Abdulnasır Yıldız, Hasan Zan

**Affiliations:** 1 Department of Electrical and Electronics Engineering, Dicle University, Diyarbakır 21200, Turkey; 2 Department of Computer Engineering, Mardin Artuklu University, Mardin 47200, Turkey; hasanzan@artuklu.edu.tr

**Keywords:** diabetic retinopathy, fundus images, patch-based learning, attention mechanism, severity grading

## Abstract

**Background/Objectives:** Automated diabetic retinopathy (DR) severity grading from fundus images is a challenging task due to the high resolution of retinal images and the spatially sparse nature of pathological lesions. **Methods:** In this study, we propose a patch-based attention framework for DR severity grading that exploits localized information while avoiding aggressive global downsampling. Each fundus image is partitioned into non-overlapping patches, which are processed using a shared ResNet-18 encoder to extract patch-level feature representations. An attention-based aggregation mechanism then assigns adaptive importance weights to the extracted features and produces an image-level representation for severity classification. The proposed framework is evaluated on the APTOS 2019 dataset. **Results:** An analysis of different patch configurations identifies 25 patches as the best-performing setting, achieving a multi-class accuracy of 0.852, compared with 0.817 for the whole-image baseline. A controlled ablation using the same effective input resolution further shows that attention-based aggregation achieves the highest accuracy and F1-score among the evaluated whole-image and patch-based aggregation configurations. The model also achieves a binary DR/No-DR accuracy of 0.986. Attention visualizations provide qualitative insight into the regions contributing to model predictions. **Conclusions:** These results demonstrate the potential of attention-guided patch-based feature learning for accurate and interpretable diabetic retinopathy severity grading.

## 1. Introduction

Diabetic retinopathy (DR) is one of the most common microvascular complications of diabetes mellitus and remains a leading cause of vision impairment and preventable blindness among the working-age population worldwide [1]. The increasing global prevalence of diabetes has led to a corresponding rise in DR cases, placing a substantial burden on healthcare systems and ophthalmology services [2]. Early detection and accurate grading of DR severity are critical for timely clinical intervention, as appropriate treatment at early stages can effectively slow disease progression and significantly reduce the risk of irreversible vision loss [3]. However, large-scale screening and continuous monitoring pose considerable challenges due to the growing number of patients requiring regular retinal examinations [4].

Color fundus photography is widely used in clinical practice as a non-invasive and cost-effective imaging modality for the screening and assessment of diabetic retinopathy [5,6]. DR manifests in fundus images through a variety of pathological signs, including microaneurysms, hemorrhages, hard exudates, and neovascularization, which typically appear in localized and spatially heterogeneous regions of the retina [7]. The visual characteristics of these lesions can vary significantly in size, shape, contrast, and distribution across disease stages, making reliable interpretation challenging, particularly in early or borderline cases [8]. Consequently, accurate analysis of fundus images requires models that are capable of capturing fine-grained local features while preserving the broader contextual information necessary for severity grading.

Traditionally, the diagnosis and grading of diabetic retinopathy from fundus images rely on manual assessment by trained ophthalmologists, a process that is time-consuming, labor-intensive, and subject to inter- and intra-observer variability [9]. These limitations become more pronounced in large-scale screening programs, where the demand for rapid and consistent evaluations often exceeds the available clinical resources [10]. To address these challenges, automated computer-aided diagnosis systems have been extensively investigated as a means to improve efficiency, consistency, and scalability in diabetic retinopathy screening.

In recent years, numerous automated approaches based on classical machine learning have been proposed for diabetic retinopathy detection and severity classification using fundus images. These methods generally rely on handcrafted feature extraction followed by conventional classifiers, such as k-nearest neighbors, support vector machines, AdaBoost, and Gaussian mixture models, to distinguish between different DR stages [11]. Several studies have focused on extracting clinically relevant features, including texture descriptors, intensity statistics, entropy measures, and vascular characteristics, which are subsequently classified using probabilistic neural networks, decision trees, or SVMs [12,13]. Segment-based learning strategies have also been explored to enhance lesion sensitivity and grading performance through dedicated diabetic retinopathy estimation classifiers [14].

Other machine-learning-based studies have emphasized explicit lesion and structure detection to support DR classification. These include approaches targeting exudates through pathological pattern mining combined with contextual information [15], as well as hybrid frameworks integrating fuzzy image processing, circular Hough transforms, and multiple ML classifiers to improve robustness [16]. In addition, feature-based methods focusing on retinal structures such as blood vessels, optic discs, exudates, and microaneurysms—often extracted using morphological operations or matched filtering—have been applied to detect mild and moderate DR stages [17,18]. Despite their effectiveness, these methods remain highly dependent on handcrafted features and carefully designed preprocessing pipelines, which can limit their scalability and generalization to diverse imaging conditions.

Deep learning–based techniques have become the dominant paradigm for automated diabetic retinopathy detection and severity classification from fundus images. Several studies have demonstrated the effectiveness of deep convolutional neural networks in learning hierarchical representations directly from retinal images, enabling multi-class DR classification across different severity levels [19]. Subsequently, a variety of well-established CNN architectures, including VGG, ResNet, GoogLeNet, DenseNet, Inception, and their variants, have been widely adopted for tasks such as DR classification, lesion detection, and segmentation, either through end-to-end training or transfer learning from large-scale natural image datasets [20,21]. These approaches have generally demonstrated strong performance while alleviating the need for handcrafted feature design and enabling the learning of more discriminative visual representations.

Various architectural refinements and preprocessing strategies have also been explored to enhance DR grading performance. EfficientNet-based models have been applied for DR severity classification and lesion detection, sometimes in combination with explicit preprocessing and morphological operations to emphasize red and white lesions [22]. Other approaches incorporate dimensionality reduction techniques, such as principal component analysis and singular value decomposition, to address the high dimensionality of deep feature representations before classification [23]. Ensemble and hybrid frameworks, including the stacking of multiple CNN backbones and the integration of spatial pyramid pooling or network-in-network layers, have been proposed to improve robustness across multiple DR classes [24,25,26].

Despite the significant progress achieved by both machine learning– and deep learning–based approaches, several important challenges remain unaddressed in automated diabetic retinopathy severity grading. Most existing deep learning methods rely on globally resized fundus images to fit the input constraints of standard CNN architectures, which can lead to the loss of fine-grained lesion information that is critical for accurate severity assessment [27,28]. Although some studies incorporate lesion-aware preprocessing or multi-scale representations, they often treat all image regions equally or depend on heuristic aggregation strategies, limiting their ability to selectively emphasize diagnostically relevant retinal areas [29]. Furthermore, fundus images are characterized by high resolution and spatially sparse pathological patterns, yet many models are not explicitly designed to handle this imbalance between informative and non-informative regions.

Patch-based and multiple-instance-learning approaches have previously been investigated for retinal image analysis. For example, Papadopoulos et al. [30] employed a multiple-instance-learning framework in which retinal patches were independently encoded and subsequently aggregated using an attention mechanism for referable DR detection. These studies demonstrate the potential of localized patch representations and attention-based aggregation for retinal image analysis. However, the use of such representations for fine-grained five-class DR severity grading remains less systematically explored, particularly with respect to how patch configuration, spatial information, feature extraction architecture, and aggregation strategy influence classification performance. Referable DR detection addresses a different classification objective from severity grading, where the model must distinguish among multiple disease stages with potentially subtle differences in retinal appearance. This motivates a systematic investigation of patch-based representations and aggregation mechanisms specifically for five-class DR severity classification.

To address these limitations, this study develops a patch-based attention framework for automated diabetic retinopathy severity grading using fundus images. Each high-resolution fundus image is partitioned into non-overlapping patches, enabling localized feature extraction while reducing the need for aggressive global downsampling. A shared convolutional patch encoder is employed to extract feature representations from individual patches, while learnable positional embeddings preserve information about their spatial arrangement. An attention-based aggregation mechanism then assigns adaptive importance to the position-aware patch representations and integrates them into an image-level representation for five-class DR severity classification. Beyond introducing this framework, the study systematically examines the factors that influence its performance, including the number of patches, the patch encoder architecture, and the aggregation strategy. The main contributions of this study are as follows:

- A high-resolution patch-based framework for five-class DR severity grading that uses non-overlapping patches to preserve localized retinal information;
- The integration of a shared patch encoder, learnable positional embeddings, and attention-based aggregation to construct position-aware image-level representations;
- A systematic investigation of the effect of the number of patches on classification performance;
- An experimental analysis of alternative patch encoder architectures to examine the dependence of the framework on the selected encoder;
- An ablation study comparing whole-image processing with patch-based representations using different aggregation strategies, including mean pooling, max pooling, and attention-based aggregation.

The remainder of this paper is organized as follows. Section 2 describes the dataset used in this study along with the experimental setup. Section 3 presents the proposed patch-based attention framework in detail, including the preprocessing steps, model architecture, and training strategy. The experimental results and their analysis are reported and discussed in Section 4. Finally, Section 5 concludes the paper and outlines potential directions for future research.

## 2. Dataset

**Figure 1:**
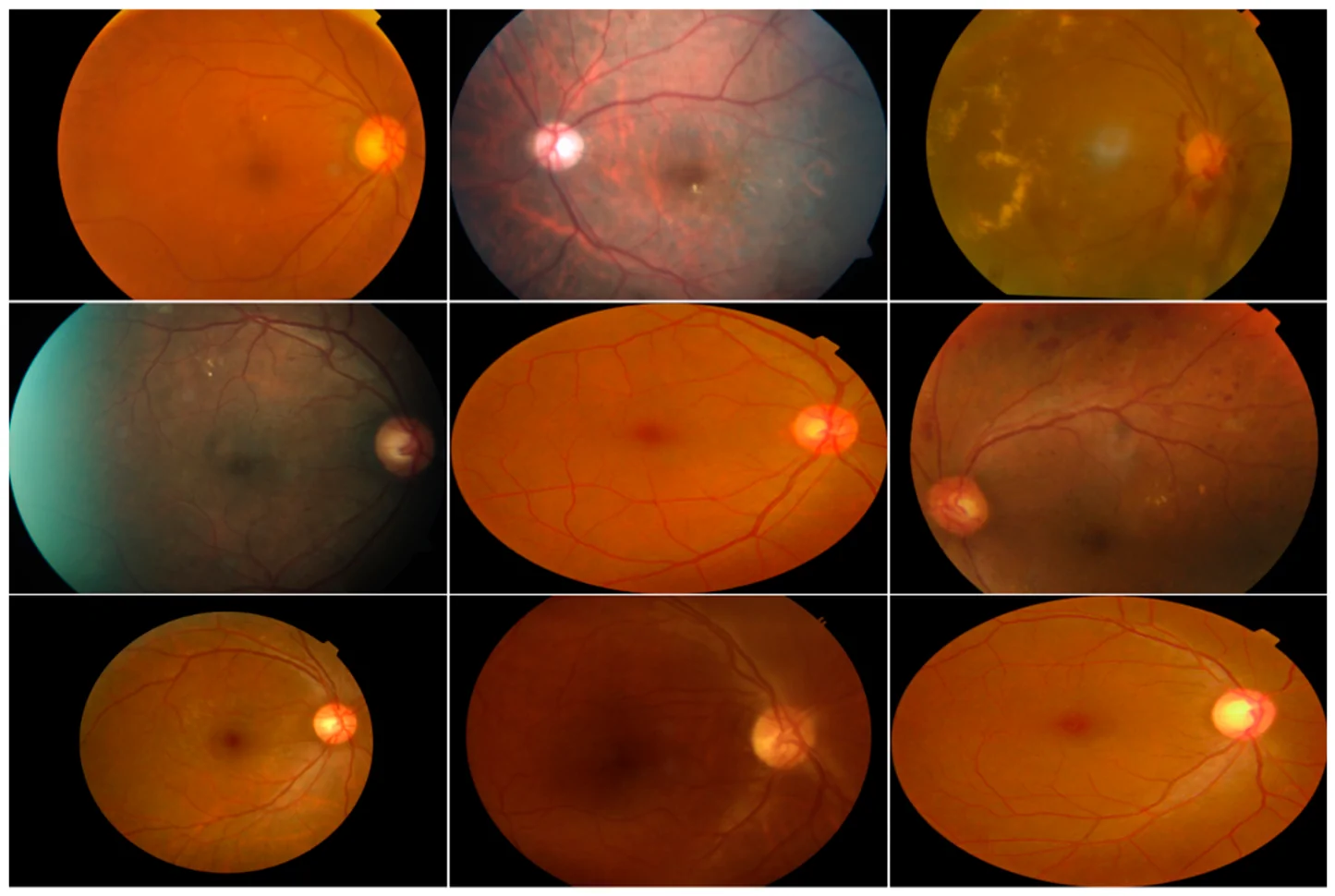
Representative fundus images from the APTOS 2019 dataset.

This study employs the Asia Pacific Tele-Ophthalmology Society (APTOS) 2019 Blindness Detection dataset, which was released as part of a public challenge aimed at advancing automated diabetic retinopathy analysis using fundus images [31]. The dataset consists of high-resolution color fundus photographs acquired under diverse imaging conditions, reflecting real-world variability in illumination, contrast, focus, and field of view. The diversity of imaging conditions provides a challenging setting for evaluating automated diabetic retinopathy severity grading methods. Representative fundus images illustrating different diabetic retinopathy severity levels are shown in Figure 1.

**Figure 2:**
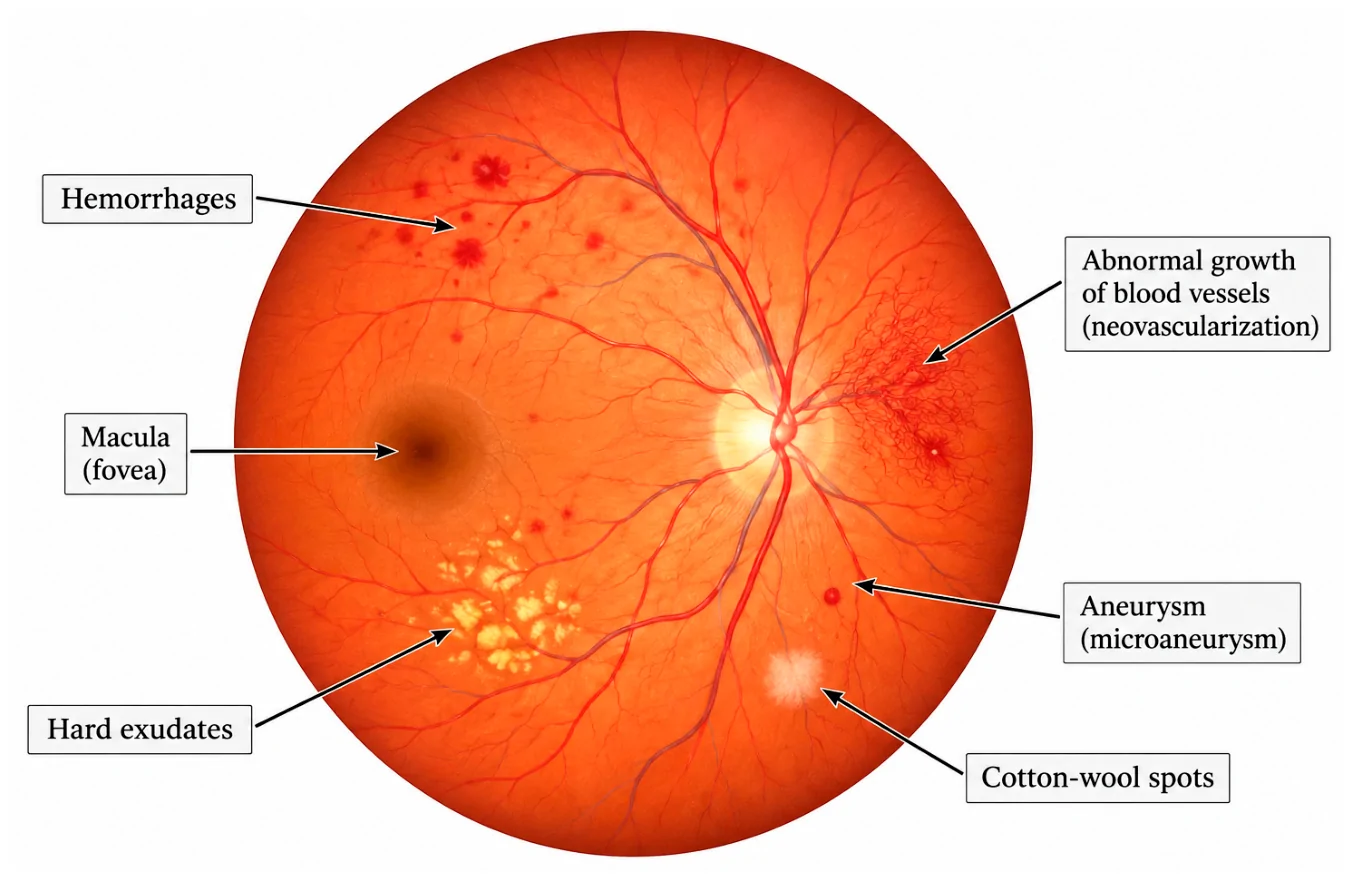
Schematic illustration of representative retinal manifestations associated with diabetic retinopathy, including hemorrhages, hard exudates, microaneurysms, cotton-wool spots, and abnormal growth of blood vessels (neovascularization).

Diabetic retinopathy is a progressive retinal microvascular disease associated with chronic hyperglycemia and can produce a range of structural and vascular changes that become increasingly pronounced with disease severity. Figure 2 provides a schematic illustration of representative retinal findings associated with diabetic retinopathy, including hemorrhages, microaneurysms, hard exudates, cotton-wool spots, and abnormal blood vessel growth. These retinal manifestations form the visual basis for distinguishing the different stages of diabetic retinopathy in fundus photographs.

In the absence of clinically visible DR, the retina generally exhibits no characteristic pathological lesions and is therefore categorized as No DR. The initial stage, Mild DR, is typically characterized by the appearance of microaneurysms, which represent localized abnormalities of retinal capillaries and are among the earliest clinically observable manifestations of the disease. As the disease progresses to Moderate DR, additional retinal abnormalities may develop, including increasing numbers of microaneurysms and retinal hemorrhages, together with lesions such as hard exudates, cotton-wool spots, venous abnormalities, and intraretinal microvascular abnormalities. These changes reflect increasing retinal vascular damage and disruption of normal retinal perfusion [32].

In Severe DR, retinal vascular abnormalities become more extensive and widespread. Multiple hemorrhages and microaneurysms, venous beading, and intraretinal microvascular abnormalities may occur across multiple retinal regions. At this stage, the retinal circulation is substantially compromised, increasing the risk of progression to the proliferative stage [32]. Proliferative DR represents the advanced stage of the disease and is characterized primarily by retinal neovascularization, in which abnormal and fragile new blood vessels develop in response to retinal ischemia. These vessels can subsequently cause preretinal or vitreous hemorrhages and may contribute to fibrous tissue formation, retinal traction, and severe visual impairment. Thus, the five severity categories represent a progression from the absence of visible retinal pathology to increasingly extensive microvascular damage and, ultimately, pathological neovascularization [33]. These progressive changes provide the clinical basis for the five-class severity labels used in the APTOS 2019 dataset.

Each fundus image in the dataset is annotated with a severity label corresponding to the clinically recognized stages of diabetic retinopathy, namely No DR, Mild, Moderate, Severe, and Proliferative DR. In this study, we use the same training, validation, and test splits employed by [29], which were generated using a stratified data split with a ratio of 80:10:10. These splits contain 2930, 366, and 366 images, respectively. The distribution of images across severity classes for each subset is summarized in Table 1, highlighting the inherent class imbalance present in the dataset. The filenames corresponding to each subset are provided in [34] to facilitate reproducibility of the experimental setup.

## 3. Methodology

This section presents the proposed computational pipeline for automated diabetic retinopathy severity grading using fundus images. It outlines the overall patch-based attention framework, including the image partitioning strategy, patch-level feature extraction, attention-based aggregation, and the deep learning components employed for model training and evaluation.

### 3.1. Overview

**Figure 3:**
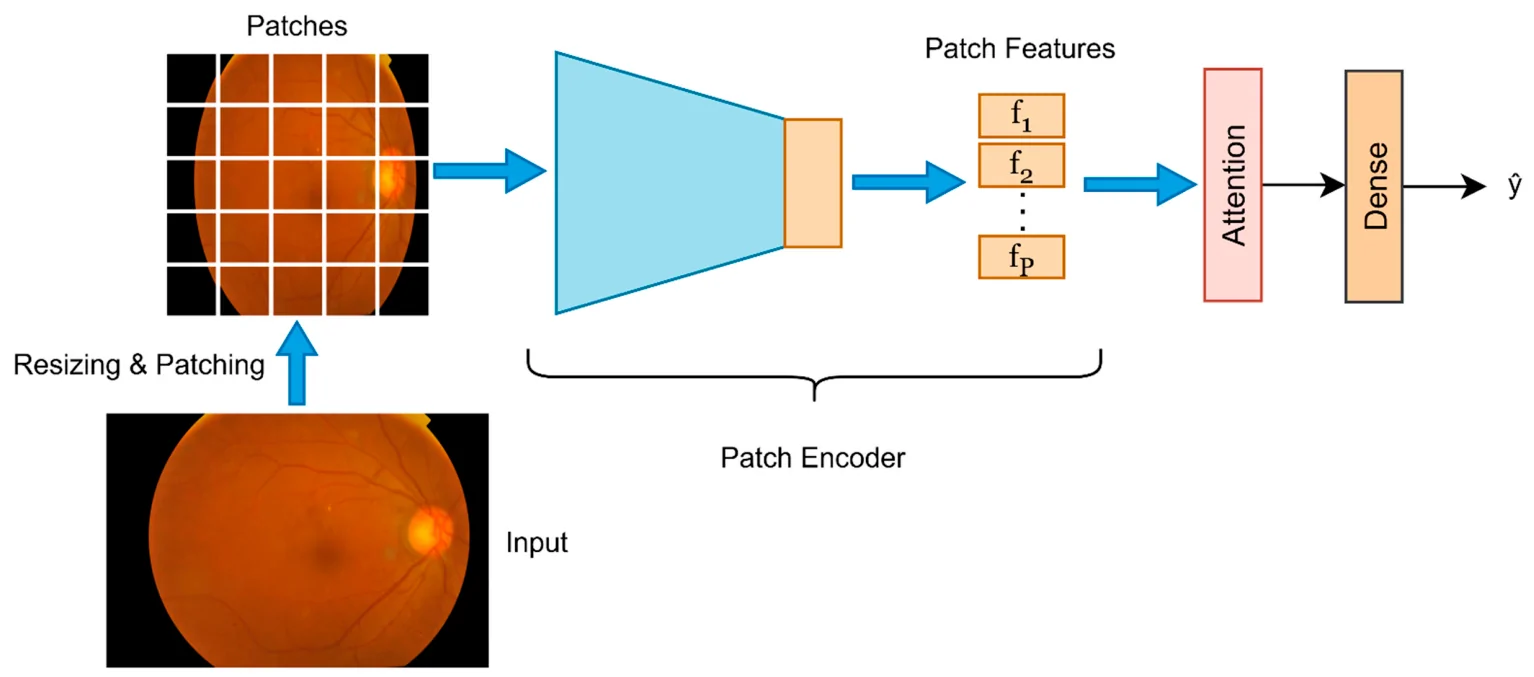
Block diagram of the proposed patch-based attention framework for automated diabetic retinopathy severity grading using fundus images.

The proposed framework is designed to address the challenges of diabetic retinopathy detection and severity grading from high-resolution fundus images, where subtle pathological cues such as microaneurysms and exudates may be lost when images are aggressively resized. To overcome this limitation, a patch-based attention framework is adopted, enabling localized analysis while preserving fine-grained retinal structures. As illustrated in Figure 3, each fundus image is resized such that its spatial dimensions are exact multiples of the predefined patch size, allowing the image to be evenly partitioned into non-overlapping patches without introducing padding artifacts.

After patch extraction, each patch is independently augmented to improve robustness against acquisition variability and local distortions. The augmented patches are then processed by a shared convolutional encoder that learns discriminative feature representations in a parameter-efficient manner. To integrate patch-level features into an image-level prediction, an attention-based aggregation mechanism is employed, enabling the model to assign adaptive importance weights to patches that contain diagnostically relevant lesions. The resulting attention-weighted representation is subsequently passed through classification layers to predict the severity stage of diabetic retinopathy. This hierarchical design allows the framework to capture both localized lesion patterns and global contextual information, resulting in a robust and interpretable approach to DR severity grading.

### 3.2. Proposed Model

The proposed model is composed of three main components: a patch encoder, an attention aggregator, and a classification head, which together form an end-to-end framework for diabetic retinopathy severity grading. This modular design enables effective learning from high-resolution fundus images while maintaining computational efficiency and interpretability.

Let an input fundus image be denoted as **I** ∈ ℝ*^H^*^×*W*×3^. The image is first resized so that its spatial dimensions are exact multiples of a predefined patch size *s*, and is then partitioned into *P* non-overlapping patches as

**I** → {**p_1_**, **p_2_**,…, **p_P_** },        **p_i_** ∈ ℝ*^s^*^×*s*×3^ (1)

This patching strategy enables localized analysis of retinal regions while preserving fine-grained pathological details that may be lost under global resizing.

Each patch **p***_i_* is processed independently by a shared patch encoder based on ResNet-18. The encoder extracts discriminative features while maintaining parameter efficiency through weight sharing across all patches. Formally, the patch encoder maps each patch to a *D*-dimensional feature vector as

**f_i_** = ℇ(**p_i_** ),        **f_i_** ∈ ℝ*^D^* (2)

where ℰ(⋅) denotes the ResNet-18 feature extractor. The resulting patch-level features are stacked to form a feature matrix,

**F** = [**f_1_**, **f_2_**,…,**f_P_**] ∈ ℝ*^P^*^×*D*^ (3)

To preserve the spatial position of each patch, a learnable positional embedding is added to the corresponding patch-level feature before attention-based aggregation. Specifically, a learnable positional embedding matrix **E**_pos_ ∈ ℝ*^P^*^×*D*^ is added to the feature matrix as

**F′** = **F** + **E_pos_** (4)

The resulting position-aware patch features are then provided to the attention-based aggregation mechanism. This module learns to assign adaptive importance weights to each patch according to its relevance for diabetic retinopathy severity grading. For each patch feature **f′_i_**, an attention score *a_i_* is computed using a lightweight feedforward network,

(5) $$a_{i}=w_{2}^{⊤}\sigma \left(W_{1}{f^{\prime }}_{i}\right)$$

where **W**_1_ and **w**_2_ are learnable parameters and *σ*(⋅) denotes the ReLU activation. For the ResNet-18 patch encoder, the input feature dimension is 512, and the hidden dimension of the attention network is 256. The lightweight two-layer formulation is adopted to provide a simple and parameter-efficient mechanism for learning patch importance weights while preserving the feature dimensionality of the position-aware patch representations. The attention scores are normalized across all patches using a softmax function,

(6) $$\alpha_{i}=\frac{\exp\left(a_{i}\right)}{\sum_{j=1}^{P}\exp(a_{j})}$$

yielding attention weights α_i_ that reflect the diagnostic importance of each patch. The final image-level representation is obtained as a weighted sum of patch features,

(7) $$z=\sum_{i=1}^{P}\alpha_{i}{f^{\prime }}_{i},\;\;\;\;\;\;z\in R^{D}$$

This mechanism allows the model to assign greater importance to informative patch representations while reducing the contribution of less relevant regions.

Finally, the classification head maps the aggregated image-level representation **z** to the predicted diabetic retinopathy severity. This component consists of fully connected layers followed by a softmax activation function,

(8) $$\hat{y}=\mathrm{softmax}\left(W_{c}z+b_{c}\right)$$

where $\hat{y}\in R^{C}$ represents the predicted probability distribution over *C* classes. The entire model is trained end-to-end, enabling the patch encoder, attention aggregator, and classification head to be jointly optimized for accurate and interpretable diabetic retinopathy severity grading.

### 3.3. Data Augmentation

To enhance the generalization capability of the proposed framework and mitigate overfitting arising from the limited size and class imbalance of the APTOS 2019 dataset, a two-stage data augmentation strategy is adopted during training. This strategy comprises global image-level transformations applied prior to patch extraction, followed by patch-level augmentations applied independently to each extracted patch. For validation and testing, the same preprocessing pipeline is used without stochastic augmentations to ensure fair and consistent evaluation.

At the global level, each fundus image is first converted to a tensor and resized to a spatial resolution of (*sN*) × (*sN*), where *s* denotes the patch size and *N* represents the number of patches along each spatial dimension. During training, random horizontal and vertical flipping and random rotation of up to 10° are applied to the entire resized image before patch extraction. These global geometric transformations introduce variations in image orientation while maintaining the spatial arrangement of retinal structures within the transformed image. The transformed image is subsequently converted to a floating-point representation and normalized using the ImageNet mean and standard deviation values. For validation and testing, no stochastic global transformations are applied; images are only resized, converted to floating-point representations, and normalized.

**Figure 4:**
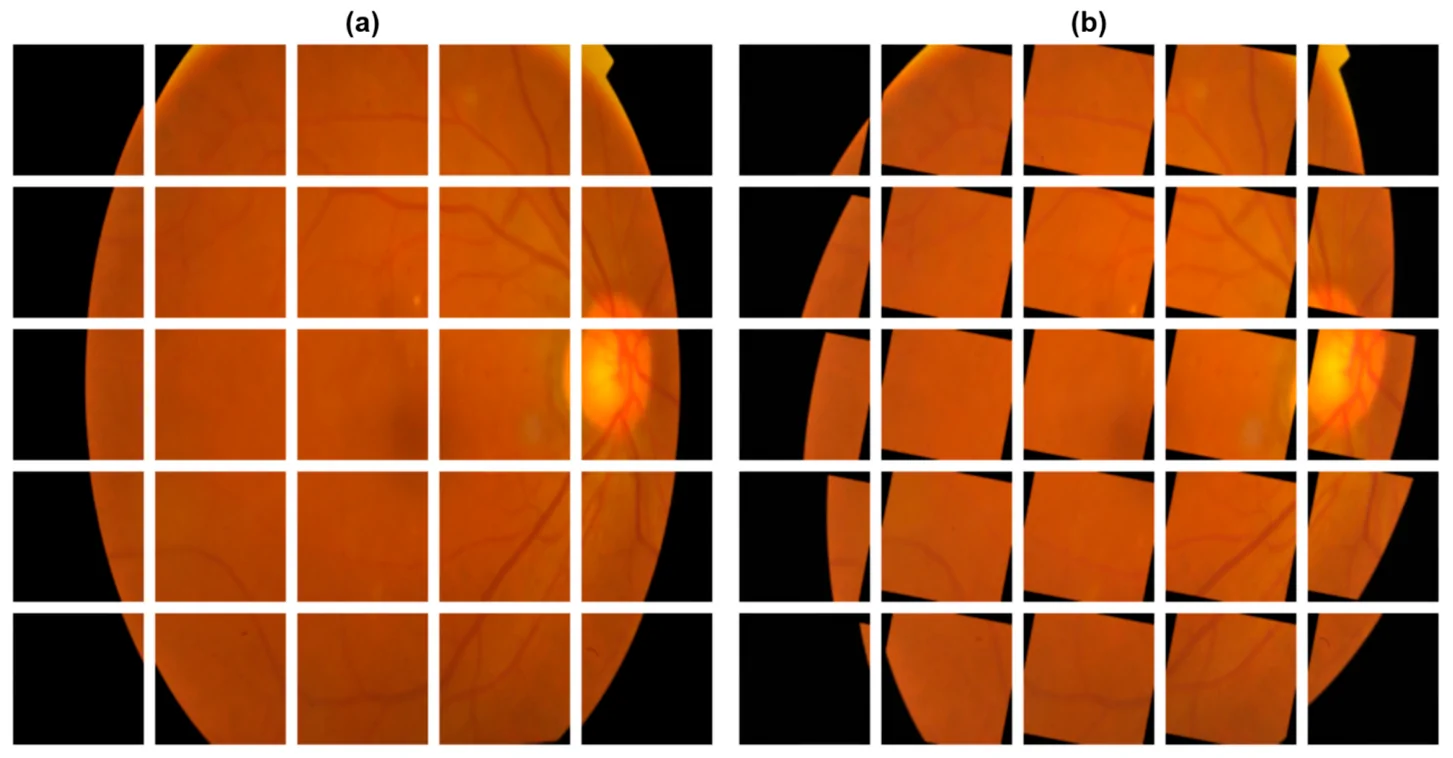
Illustration of the two-stage data augmentation process: (**a**) non-overlapping patches extracted from a resized fundus image and (**b**) the corresponding patches after the augmentation pipeline is applied during training.

Following global preprocessing, each resized image is partitioned into *P* = *N*^2^ non-overlapping patches, with each patch having a spatial resolution of *s* × *s*. During training, additional patch-level augmentation is applied independently to the extracted patches using probabilistic ColorJitter and Gaussian blurring. Specifically, ColorJitter is applied with a probability of 0.5, while Gaussian blurring is applied with a probability of 0.3. These transformations introduce localized photometric variations and increase the diversity of patch-level feature representations without independently flipping or rotating neighboring patches. The augmented patches are then processed by the shared pretrained ResNet-18 patch encoder to extract local feature representations. Learnable positional embeddings are subsequently added to these features before attention-based aggregation, allowing the model to incorporate information about the spatial arrangement of the patches. This two-stage augmentation strategy combines global geometric transformations with localized appearance variations. The process is illustrated in Figure 4, which presents representative input patches alongside their augmented counterparts.

### 3.4. Model Training

The proposed model is trained in an end-to-end manner using the APTOS 2019 dataset. The patch configuration and other model settings are selected based on performance on the validation set, while early stopping is also guided by validation accuracy. In particular, the 25-patch configuration is selected based on its validation performance, and the test set is reserved for the final evaluation of the selected configuration. During training, the data augmentation strategies described in Section 3.3 are applied to improve generalization and robustness to variations in image acquisition and local appearance. The patch encoder is initialized with the default ImageNet-pretrained weights of ResNet-18. For the experiments using 25 patches, each patch has a spatial resolution of 224 × 224 pixels, resulting in an effective input image resolution of 1120 × 1120 pixels before patch extraction.

Optimization is performed using the Adam optimizer [35] with an initial learning rate of 5 × 10^−4^. No learning-rate scheduler is used during training. The weight decay parameter is set to the default value of the PyTorch Adam optimizer. The model is trained for a maximum of 100 epochs, with early stopping employed to prevent overfitting. Specifically, training is terminated if the validation accuracy does not improve for 20 consecutive epochs, and the model parameters corresponding to the best validation accuracy are retained. A batch size of 16 is used throughout training.

To address the pronounced class imbalance in the training data, a weighted cross-entropy loss function is adopted. The class weights are calculated from the class frequencies in the training set according to *w_c_* = *N*/*n_c_*, where *N* denotes the total number of training samples and *n_c_* denotes the number of samples belonging to class *c*. The resulting weights are subsequently normalized such that their sum equals 100. This weighting strategy increases the contribution of underrepresented severity classes during optimization and reduces the influence of the majority classes.

To improve reproducibility, the random seeds for PyTorch, CUDA, NumPy, and Python’s built-in random module are set to 0. All experiments are conducted using a single NVIDIA A100 GPU with 80 GB of memory and an Intel Xeon Gold 8362 processor operating at 2.80 GHz. The implementation uses Python 3.12 and PyTorch 2.2.

**Figure 5:**
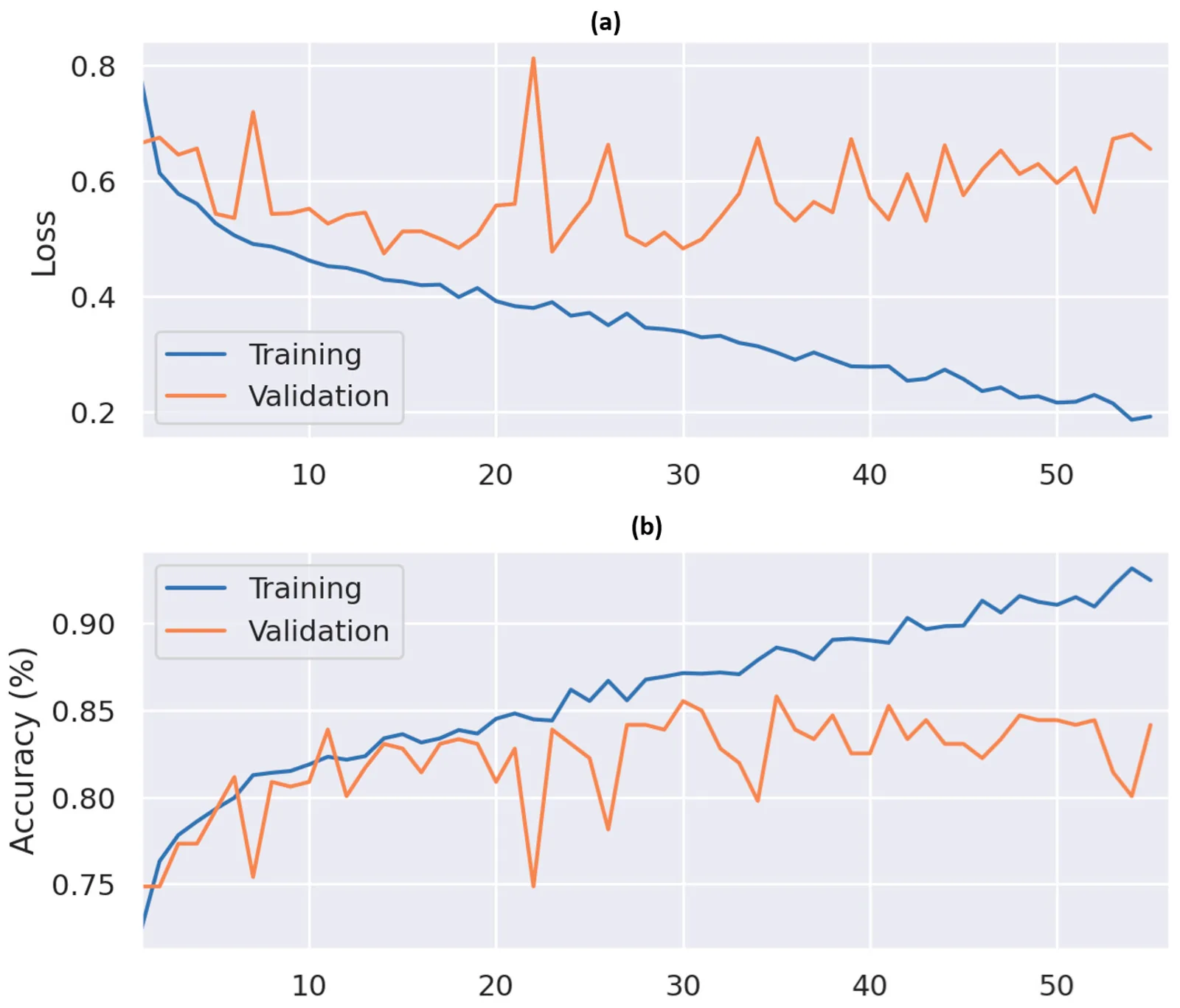
Training dynamics of the proposed model: (**a**) training and validation losses across epochs, and (**b**) training and validation accuracies across epochs.

The evolution of the training and validation losses and accuracies throughout the optimization process is illustrated in Figure 5, providing insight into the convergence behavior and generalization performance of the proposed framework.

### 3.5. Model Evaluation

The performance of the proposed framework is evaluated using both class-wise and overall metrics to provide a comprehensive assessment of diabetic retinopathy (DR) severity grading. Given the inherent class imbalance in the APTOS 2019 dataset and the importance of accurately identifying each DR severity stage, class-wise precision, recall, and F1-score are reported alongside overall classification accuracy. For the overall precision, recall, and F1-score, the macro-average across the five severity classes is used, giving equal weight to each class regardless of its frequency.

For a given DR severity class *c*, let *TP_c_*, *FP_c_*, and *FN_c_* denote the numbers of true positives, false positives, and false negatives, respectively. Precision measures the reliability of positive predictions for class *c* and is defined as

(9) $${\mathrm{Precision}}_{c}=\frac{TP_{c}}{TP_{c}+FP_{c}}$$

Recall, also referred to as sensitivity, quantifies the ability of the model to correctly identify samples belonging to class *c* and is given by

(10) $${\mathrm{Recall}}_{c}=\frac{TP_{c}}{TP_{c}+FN_{c}}$$

The F1-score provides a balanced measure by harmonically combining precision and recall and is computed as

(11) $${F1}_{c}=\frac{2\cdot {\mathrm{Precision}}_{c}\cdot {\mathrm{Recall}}_{c}}{{\mathrm{Precision}}_{c}+{\mathrm{Recall}}_{c}}$$

To obtain overall class-balanced measures, the macro-averaged precision, recall, and F1-score are calculated as the arithmetic mean of the corresponding class-wise metrics:

(12) $$\mathrm{Macro}\text{-}\mathrm{Precision}=\frac{1}{C}\sum_{c=1}^{C}{\mathrm{Precision}}_{c}$$

(13) $$\mathrm{Macro}\text{-}\mathrm{Recall}=\frac{1}{C}\sum_{c=1}^{C}{\mathrm{Recall}}_{c}$$

(14) $$\mathrm{Macro}\text{-}F1=\frac{1}{C}\sum_{c=1}^{C}{F1}_{c}$$

where *C* denotes the number of DR severity classes. Macro-averaging assigns equal importance to all classes and is therefore particularly informative for the imbalanced class distribution of the evaluated dataset.

In addition to macro-averaged metrics, the weighted F1-score is reported to account for the relative frequency of each severity class. It is calculated as

(15) $$\mathrm{Weighted}\text{-}F1=\sum_{c=1}^{C}\frac{N_{c}}{N}{F1}_{c}$$

where *N_c_* denotes the number of samples belonging to class *c* and *N* is the total number of samples in the evaluation set.

Overall classification accuracy is used to summarize the proportion of correctly classified samples across all classes and is defined as

(16) $$\mathrm{Accuracy}=\frac{\sum_{c=1}^{C}TP_{c}}{N}$$

To further account for class imbalance, balanced accuracy is calculated as the arithmetic mean of the recall values across all severity classes:

(17) $$\mathrm{Balanced Accuracy}=\frac{1}{C}\sum_{c=1}^{C}{\mathrm{Recall}}_{c}$$

The Matthews correlation coefficient (MCC) is also reported as a measure of the agreement between the predicted and true class assignments. For multiclass classification, MCC is calculated from the complete confusion matrix, thereby incorporating information from all classes simultaneously. Its value ranges from −1 to 1, with higher values indicating stronger agreement between predictions and ground-truth labels.

Because DR severity grades represent an ordinal classification problem, quadratic weighted kappa (QWK) is additionally reported. Unlike conventional accuracy-based measures, QWK accounts for the magnitude of disagreement between predicted and true severity levels by assigning larger penalties to predictions that are farther from the correct class. Thus, QWK provides a complementary assessment of model performance for the ordered DR severity categories.

Finally, a complementary binary evaluation is performed by combining the Mild, Moderate, Severe, and Proliferative DR classes into a single DR category and evaluating the model against the No DR class. This analysis provides an additional assessment of the model’s ability to distinguish images with DR from those without DR.

Together, these evaluation metrics provide complementary perspectives on model performance, including class-specific discrimination, class-balanced performance, overall predictive accuracy, agreement across all classes, and the ordinal nature of DR severity grading. This combination is intended to provide a more comprehensive evaluation than accuracy alone, particularly in the presence of the class imbalance characteristic of the APTOS 2019 dataset.

## 4. Results and Discussion

This section presents a comprehensive evaluation of the proposed patch-based attention framework for diabetic retinopathy severity grading using fundus images. Quantitative results are reported on the test set following the evaluation protocol described in Section 3.5. The performance of the model is first analyzed at the class level to examine its ability to distinguish different DR severity stages. Overall performance is then evaluated using aggregate classification metrics, including accuracy, macro-averaged and weighted metrics, balanced accuracy, Matthews correlation coefficient (MCC), and quadratic weighted kappa (QWK). In addition, the results are examined in relation to the design choices of the proposed framework, with particular emphasis on the effect of patch-based representation, the number of patches, the patch encoder architecture, and the patch aggregation strategies.

### 4.1. Class-Based Performance

**Figure 6:**
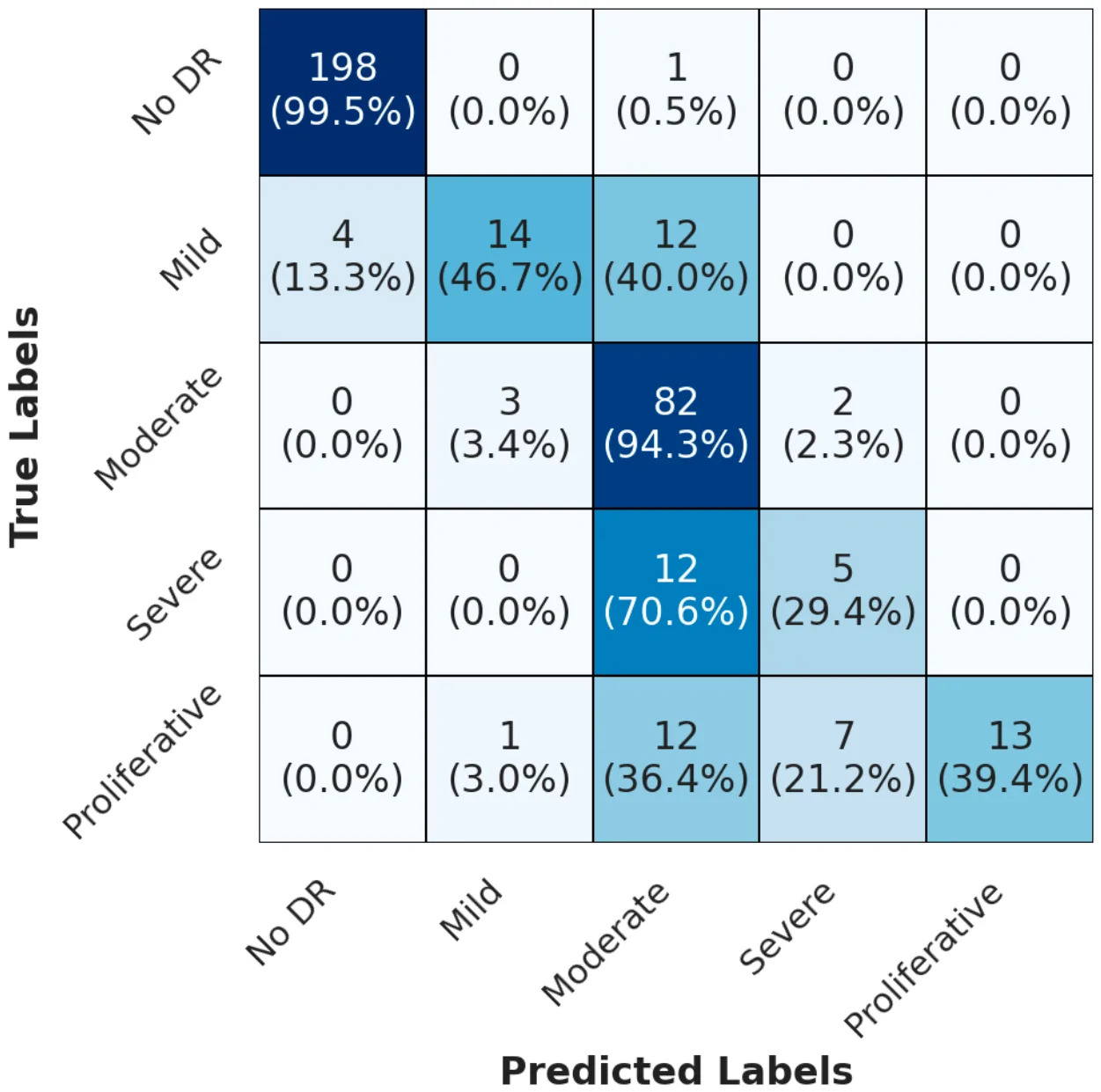
Confusion matrix of the proposed model on the test set, illustrating class-wise prediction counts and corresponding percentages for diabetic retinopathy severity grading. The color intensity represents the number of samples in each cell, with darker shades indicating higher counts and lighter shades indicating lower counts.

The class-wise performance of the proposed framework using *P* = 25 patches on the test set is presented in Table 2, while the corresponding confusion matrix is shown in Figure 6. The model achieves an overall classification accuracy of 0.852, while the class-wise results demonstrate variation in performance across the five DR severity levels.

The model demonstrates particularly strong performance for the No DR class, achieving a precision of 0.980, recall of 0.995, and F1-score of 0.988. As illustrated in Figure 6, 198 out of 199 No DR samples are correctly classified, with only one sample misclassified as Moderate DR. This high level of performance indicates that the model can reliably distinguish images without visible DR-related severity from the diseased classes in the evaluated test set. Such performance is particularly relevant to screening-oriented applications, where accurate identification of non-DR cases can help reduce unnecessary referrals.

For the Moderate DR class, the proposed method achieves a high recall of 0.943 and an F1-score of 0.796, with 82 out of 87 samples correctly classified. Most of the remaining Moderate cases are classified as Mild, Severe, or Proliferative DR, indicating that the model generally recognizes Moderate DR effectively while occasionally confusing it with neighboring severity levels. The strong performance of this class may suggest that the patch-based representation provides useful localized features for distinguishing the characteristic retinal patterns present in Moderate DR.

In contrast, the Mild and Severe classes exhibit comparatively lower performance. The Mild DR class achieves a precision of 0.778, recall of 0.467, and F1-score of 0.583. Of the 30 Mild DR samples, 14 are correctly classified, while 12 are classified as Moderate DR and four as No DR. The relatively low recall indicates difficulty in consistently distinguishing Mild DR from other severity levels, particularly Moderate DR. This pattern may reflect the visual similarity between early-stage pathological changes and more pronounced manifestations of Moderate DR. Similarly, the Severe DR class presents greater classification difficulty, achieving a precision of 0.357, recall of 0.294, and F1-score of 0.323. Only 5 of the 17 Severe DR samples are correctly classified, while 12 are predicted as Moderate DR. This substantial concentration of errors between Severe and Moderate DR indicates that distinguishing these adjacent severity levels remains challenging for the proposed framework.

For the Proliferative DR class, the model achieves a precision of 1.000, recall of 0.394, and F1-score of 0.565. Of the 33 Proliferative DR samples, 13 are correctly classified, whereas 12 are assigned to Moderate DR, seven to Severe DR, and one to Mild DR. The perfect precision indicates that every prediction assigned to the Proliferative DR class corresponds to an actual Proliferative DR sample in the evaluated test set. However, the relatively low recall shows that a substantial proportion of actual Proliferative DR cases are assigned to lower severity categories. This pattern further highlights the difficulty of distinguishing advanced DR stages when pathological characteristics may overlap across neighboring severity levels.

Overall, the class-wise results show that the model performs particularly well for No DR and Moderate DR, whereas Mild, Severe, and Proliferative DR remain more challenging. Most errors occur between neighboring severity levels, particularly between Mild and Moderate DR and between Severe and Moderate DR. This pattern suggests that fine-grained severity discrimination remains more challenging than the recognition of clearly distinguishable classes.

### 4.2. Overall Performance

The overall performance of the proposed framework is assessed using several complementary metrics. Using *P* = 25 patches, the model correctly classifies 312 of the 366 test samples, resulting in an overall accuracy of 0.852. The macro-averaged precision, recall, and F1-score are 0.761, 0.618, and 0.651, respectively. Because macro-averaging assigns equal importance to each severity class, these metrics provide a balanced assessment of classification performance under the imbalanced class distribution of the test set. The balanced accuracy of 0.618 further reflects the average recall across the five severity classes, while the MCC of 0.768 provides a correlation-based measure of agreement between the predicted and true class assignments.

The model also achieves a weighted F1-score of 0.840, which is higher than the macro F1-score because the weighted metric is influenced more strongly by the relatively well-represented No DR and Moderate DR classes. In addition, the proposed framework obtains a QWK of 0.912. Since DR severity levels have an ordinal structure, QWK provides a complementary assessment by accounting for the degree of disagreement between predicted and true severity levels. The relatively high QWK is consistent with the confusion matrix in Figure 6, where many misclassifications occur between neighboring severity categories rather than between widely separated classes. Together, these results indicate that the framework achieves strong overall classification performance while the macro-averaged metrics highlight the remaining difficulty in recognizing less frequent and more challenging DR severity classes.

A complementary binary evaluation was also performed by combining Mild, Moderate, Severe, and Proliferative DR into a single DR category. Based on the confusion matrix, 198 of the 199 No DR samples are correctly identified, while 163 of the 167 DR samples are correctly classified. This results in one false positive and four false negatives, yielding a binary classification accuracy of 0.986. The corresponding binary precision, recall, and F1-score are 0.994, 0.976, and 0.985, respectively. The higher binary performance compared with the five-class accuracy indicates that a substantial proportion of classification errors arises from confusion among different DR severity levels rather than from confusion between No DR and DR. However, this high binary accuracy should not be interpreted as evidence of reliable five-class severity grading. This difference reflects the distinct objectives of the two tasks: binary classification evaluates whether DR is present, whereas five-class grading additionally requires the model to distinguish among different severity levels. In particular, the misclassification of Severe and Proliferative DR cases as lower severity categories highlights the remaining difficulty in recognizing advanced disease. Therefore, the results demonstrate that fine-grained severity grading remains a major challenge for the proposed framework.

### 4.3. Attention Visualizations

**Figure 7:**
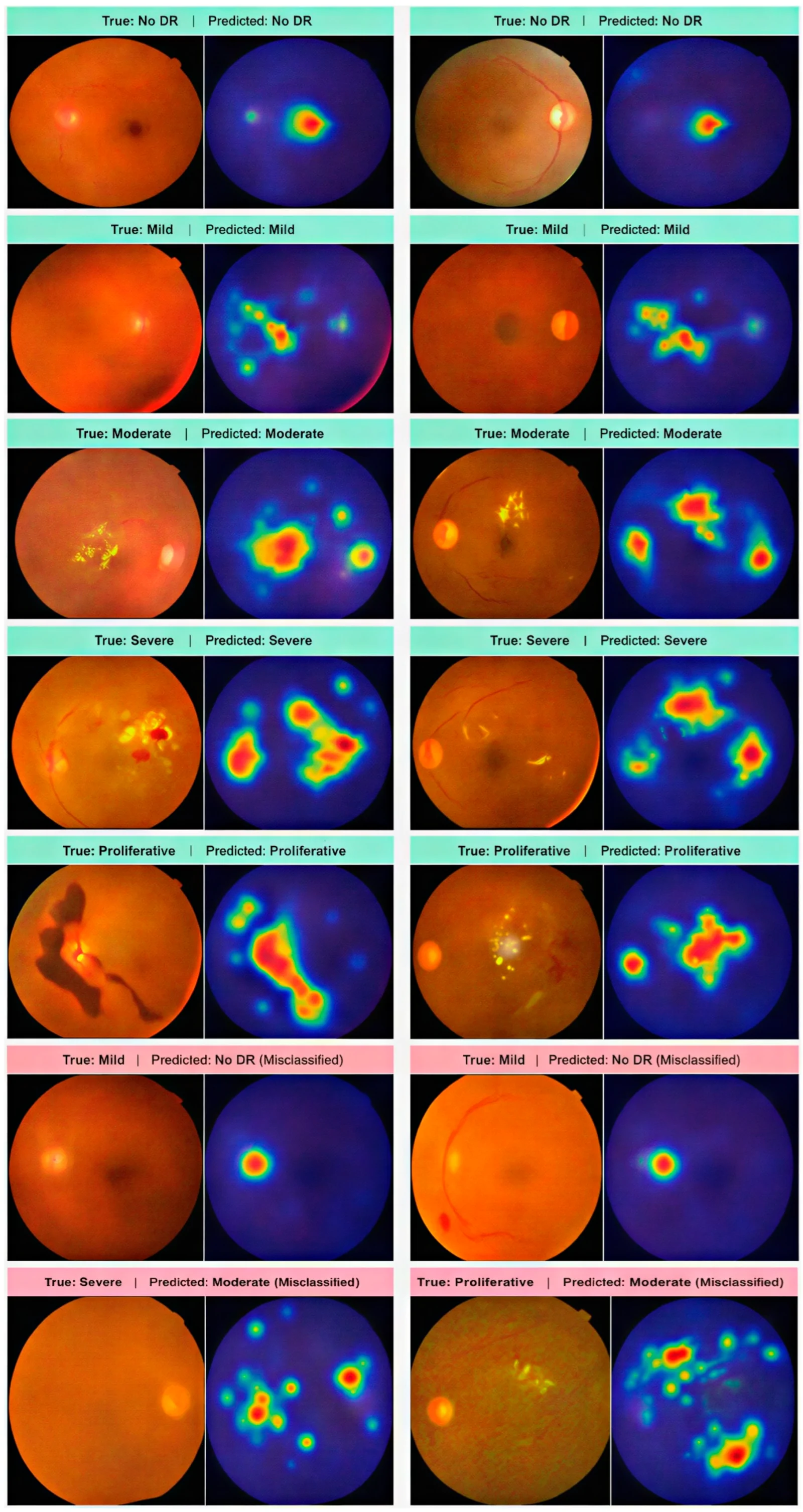
Patch-based attention visualizations of representative fundus images from the test. Each pair shows the original fundus image and the corresponding attention visualization. The attention weights are mapped to their respective spatial regions and displayed using the JET color map. Blue and cyan colors indicate lower attention weights, whereas yellow and red colors represent higher attention weights, highlighting patches assigned greater relative importance by the proposed attention mechanism.

To provide qualitative insight into the decision-making process of the proposed patch-based attention framework, representative patch-based attention visualizations from the test set are presented in Figure 7. The figure includes examples from all five DR severity categories, namely No DR, Mild, Moderate, Severe, and Proliferative DR. For each example, the original fundus image is shown alongside its corresponding attention visualization, illustrating the spatial distribution of the importance assigned to different image patches. Both correctly classified and misclassified samples are included to provide a broader view of the attention patterns across different severity levels.

The attention maps are generated from the softmax-normalized patch-level attention weights produced by the attention-based aggregation module. For the 25-patch configuration, the weights are reshaped into a 5 × 5 grid corresponding to the spatial arrangement of the patches. This grid is then bilinearly interpolated to a resolution of 1120 × 1120 pixels to produce a smooth spatial attention map. The resulting map is overlaid on the fundus image using the JET color map with an opacity of 0.3, where warmer colors indicate higher attention weights and cooler colors indicate lower weights. These visualizations illustrate the relative importance assigned to different patch locations by the model; they should not be interpreted as lesion segmentation maps.

For the correctly classified No DR examples, the attention maps show concentrated responses around specific retinal regions, including areas near the optic disc and other localized structures, without prominent attention to pathological features. In the Mild DR examples, attention is distributed across multiple regions of the fundus, including areas where small lesions resembling microaneurysms can be observed. The Moderate DR examples show relatively stronger attention in regions containing visible retinal abnormalities, particularly clusters of hard exudates and other localized changes. The Severe DR examples exhibit broader attention distributions across multiple retinal regions, with higher attention observed around areas containing hard exudates, hemorrhages, and cotton-wool spots. Similarly, the Proliferative DR examples show attention across multiple regions with prominent retinal abnormalities, including areas containing hemorrhagic patterns and extensive pathological changes. These observations indicate that the model can assign different levels of importance to spatially distributed patch representations rather than relying exclusively on a single image region.

Figure 7 also includes two misclassified Mild DR samples, both of which are predicted as No DR. In these cases, the attention maps are primarily concentrated around the optic disc and a limited number of other retinal regions, while relatively less attention is observed in areas containing subtle pathological changes. The relatively weak attention to these subtle abnormalities may be associated with the difficulty of distinguishing Mild DR from No DR in these examples. These cases illustrate how the attention distribution can differ in misclassified samples and provide qualitative insight into potential sources of classification error.

Overall, the visualizations provide qualitative information about the relative importance assigned to different retinal patches during image-level prediction. Across the five severity categories, the attention maps frequently highlight regions containing visible retinal abnormalities, including microaneurysms, hard exudates, hemorrhages, and cotton-wool spots, while attention is also distributed across surrounding retinal regions. This suggests that the model can integrate information from multiple spatially distributed regions when constructing the image-level representation. However, these visualizations should be regarded as qualitative representations of patch importance rather than definitive evidence of lesion localization or clinical interpretability.

### 4.4. Effect of the Number of Patches

To investigate the effect of the patch configuration on diabetic retinopathy (DR) severity grading, the performance of six configurations with different numbers of non-overlapping patches was evaluated. In all configurations, the patch size was fixed at 224 × 224 pixels. The number of patches, *P*, determines the spatial grid dimensions, with $N=\sqrt{P}$ patches along each dimension. Accordingly, each fundus image was resized to an effective input resolution of (224*N*) × (224*N*) pixels before being partitioned into patches. The effective input resolution therefore increases with the number of patches, although the dimensions of each individual patch remain unchanged. Increasing the number of patches provides a finer-grained spatial representation while allowing the image to be processed at a higher effective resolution. The optimal patch configuration, *P* = 25, was selected based on validation accuracy, independently of the test-set results. Table 3 presents the validation accuracy, test-set classification metrics, effective input resolution, and inference latency for all six configurations. These test-set comparisons are reported for descriptive purposes and were not used to select the optimal configuration.

In this experiment, *P* = 1 corresponds to the conventional image-level setting without patching, in which the entire fundus image is processed as a single input. As the number of patches increases from 1 to 4 and 9, the classification accuracy improves from 0.817 to 0.828 and 0.831, respectively. This initial improvement suggests that dividing the image into multiple regions allows the model to learn more localized retinal features and reduces the information loss associated with representing the entire fundus image as a single input. The corresponding improvements in precision and F1-score further indicate that localized feature extraction can provide more discriminative representations across DR severity classes.

With *P* = 16, the accuracy increases further to 0.833, despite a decrease in precision and a relatively modest recall. This result suggests that increasing the spatial granularity does not necessarily improve all evaluation metrics uniformly. Nevertheless, the overall accuracy indicates that a moderately fine-grained patch representation can provide useful complementary information for image-level classification.

The best performance is obtained with *P* = 25, achieving a precision of 0.761, recall of 0.618, F1-score of 0.651, and an overall accuracy of 0.852. Compared with the whole-image (*P* = 1) baseline, this corresponds to an accuracy improvement of 3.5 percentage points. The higher precision and F1-score further indicate that the 25-patch configuration provides more effective classification performance than the whole-image baseline. A plausible explanation is that this configuration offers a favorable balance between local detail and contextual information. The patches are sufficiently small to preserve localized pathological patterns while remaining large enough to retain meaningful retinal context. Furthermore, the attention aggregator can selectively emphasize informative patches and reduce the influence of less relevant regions, allowing the image-level representation to benefit from the increased spatial resolution. Because the number of patches simultaneously determines the granularity of the patch-based representation and the effective input resolution under the adopted fixed-patch-size implementation, the observed performance improvement reflects the combined effect of preserving spatial information and performing localized attention-based feature aggregation.

In contrast, further increasing the number of patches to *P* = 36 results in a decline in performance, with accuracy decreasing to 0.822 and F1-score to 0.564. This deterioration occurs even though the effective input resolution increases to 1344 × 1344 pixels. The individual patch dimensions, however, remain fixed at 224 × 224 pixels. Consequently, each patch represents a smaller proportion of the complete retinal field of view at *P* = 36 than at *P* = 25, where the image is divided into 36 rather than 25 regions. Although the complete patch grid still covers the full fundus image, each individual patch contains a smaller spatial portion of the retina and may provide less surrounding context for interpreting localized abnormalities. Diagnostically relevant structures may also extend across multiple patches, making it more challenging for the model to capture their local context through patch-level representations and attention-based aggregation. These factors may contribute to the observed performance decline, although the current results do not establish a single cause for it. Thus, increasing the number of patches beyond an appropriate level does not necessarily lead to better performance.

Inference latency was measured for each patch configuration to quantify the computational cost associated with different effective input resolutions. Before measurement, the model was placed in evaluation mode, and inference was performed with gradient computation disabled. Twenty warm-up iterations were performed before timing. Measurements were then collected over 10 complete passes through the test data loader, with CUDA synchronization used to account for asynchronous GPU execution. The measured time included data loading, preprocessing, transferring each input to the computational device and performing the model’s forward pass. For each repetition, the total measured time was divided by the number of processed images to obtain the average inference time per image. Table 3 reports the mean and standard deviation across the 10 repetitions. Inference latency increased with the number of patches, from 1.272 ms/image for *P* = 1 to 8.265 ms/image for *P* = 36. The *P* = 25 configuration required 6.700 ± 0.049 ms/image under the same benchmarking protocol.

Overall, the results demonstrate that the number of patches affects both classification performance and computational cost. Among the evaluated configurations, *P* = 25 achieved the highest test accuracy (0.852), macro-precision (0.761), and macro-F1-score (0.651), while its inference latency was higher than that of configurations with fewer patches. Increasing the number of patches beyond 25 increased inference time without improving classification performance. These findings support *P* = 25 as the best-performing configuration among those evaluated, while highlighting the trade-off between classification performance and inference cost.

### 4.5. Effect of the Patch Encoder Architecture

To investigate the effect of the patch encoder architecture on the performance of the proposed framework, additional experiments were conducted using alternative convolutional neural network architectures. All experiments were performed using *P* = 25 patches, while the patch-based representation, attention aggregation mechanism, classification head, dataset partitioning, and training protocol were kept unchanged across all experiments. Only the patch encoder was replaced. In addition to the ResNet-18 encoder used in the proposed framework, ResNet-50 and EfficientNet-B0 were considered to represent deeper and more computationally advanced architectures, respectively. MobileNetV2 was also considered as a lightweight alternative. This analysis aims to determine whether the observed performance of the framework is strongly dependent on the selected patch encoder or whether the proposed patch-based attention framework remains effective across different encoder architectures.

The results in Table 4 show that the classification performance varies across the evaluated patch encoder architectures, while the overall framework remains effective with each encoder. ResNet-18 achieves an accuracy of 0.852, with a macro-averaged precision of 0.761, recall of 0.618, and F1-score of 0.651. ResNet-50, despite having substantially more parameters than ResNet-18, achieves a lower accuracy of 0.822, with precision of 0.651, recall of 0.617, and F1-score of 0.622. This indicates that increasing the depth of the patch encoder does not necessarily result in improved image-level classification performance under the adopted experimental configuration.

EfficientNet-B0 achieves an accuracy of 0.844, with a precision of 0.669, recall of 0.614, and F1-score of 0.627. Compared with ResNet-50, EfficientNet-B0 achieves higher accuracy, Precision, and F1-score, while its Recall is slightly lower (0.614 versus 0.617). It also has fewer parameters than both ResNet-18 and ResNet-50, with 4.87 million parameters. MobileNetV2, which has the smallest number of parameters among the evaluated encoders at 3.08 million, achieves an accuracy of 0.822, precision of 0.715, recall of 0.578, and F1-score of 0.602. Its recall and F1-score are the lowest among the evaluated encoders, indicating that its performance is less balanced across classes than that of the other encoders.

Overall, these results indicate that the proposed patch-based attention framework can be implemented with different convolutional patch encoders, while the choice of encoder affects the resulting classification performance. Within the evaluated configurations, ResNet-18 provides the highest overall accuracy and the most balanced combination of precision, recall, and F1-score, whereas EfficientNet-B0 offers a compelling trade-off between performance and model size. The results also show that a deeper or more computationally advanced encoder does not necessarily provide better performance within the proposed patch-based attention framework.

### 4.6. Effect of the Patch Aggregation Strategies

To further examine the contribution of the patch-based representation and the aggregation strategy, an ablation study was conducted using four configurations, as summarized in Table 5. The first configuration processes the entire fundus image at a resolution of 1120 × 1120 pixels using an ImageNet-pretrained ResNet-18 without patch-based aggregation. The remaining configurations use the same 25-patch representation, with each patch having a resolution of 224 × 224 pixels, and differ only in the aggregation strategy. Mean pooling, max pooling, and the proposed attention-based aggregation are evaluated under the same experimental protocol.

The whole-image configuration achieves an accuracy of 0.836 and an F1-score of 0.607. Using the same effective input resolution but dividing the image into 25 patches results in an accuracy of 0.825 with mean pooling and 0.833 with max pooling. Their corresponding F1-scores are 0.619 and 0.639, respectively. The attention-based aggregation achieves the highest accuracy of 0.852 and an F1-score of 0.651 among the evaluated configurations. These results show that the aggregation strategy affects the performance of the patch-based representation, with attention-based aggregation providing the highest F1-score and accuracy in this experiment.

To further assess the computational implications of the proposed approach, inference latency was measured for the whole-image and patch-based attention configurations. The whole-image ResNet-18 model achieved a mean inference time of 6.573 ± 0.092 ms per image, whereas the *P* = 25 attention-based model required 6.700 ± 0.049 ms per image. This represents an increase of approximately 1.93% in measured inference latency. In return, the patch-based attention model improved accuracy from 0.836 to 0.852 and F1-score from 0.607 to 0.651. These results indicate that the observed classification improvements were accompanied by a relatively small increase in measured inference latency.

Overall, the results indicate that the performance differences cannot be attributed solely to the increased spatial resolution, since all configurations use the same effective (1120 × 1120) image resolution. The comparison between mean pooling, max pooling, and attention-based aggregation further demonstrates the effect of the aggregation strategy when the patch representation is kept fixed. The results support the use of attention-based aggregation for integrating the position-aware patch features in the proposed framework, with the measured latency comparison providing additional information about its computational requirements.

### 4.7. Comparison with Previous Studies

The performance of the proposed patch-based attention framework was compared with representative deep learning approaches for diabetic retinopathy severity grading using the APTOS 2019 dataset, as summarized in Table 6. The studies included in Table 6 were selected based on their use of the APTOS 2019 dataset, their focus on five-class diabetic retinopathy severity grading, and similar experimental characteristics, particularly with respect to the distribution of the severity classes and the availability of a reported accuracy metric. The reported accuracies of the selected methods range from 0.725 to 0.884, while the proposed framework achieves an accuracy of 0.852 under the experimental protocol described in this study.

Among conventional convolutional neural network approaches, Mustafa et al. [39] employed DenseNet-121 and reported an accuracy of 0.725, while Farag et al. [37] achieved an accuracy of 0.820 using DenseNet-169. Amir and Almiahi [41] reported an accuracy of 0.840 with EfficientNetB3. Attention-based approaches have also been investigated for diabetic retinopathy grading. Dixit and Jha [29] combined EfficientNetB3 with attention mechanisms, including a squeeze-and-excitation block, to enhance feature representations and reported an accuracy of 0.884. Bodapati et al. [36] proposed a Composite Gated Attention CNN and achieved an accuracy of 0.825, whereas Hannan et al. [38] combined an attention mechanism with DenseNet-169 and reported an accuracy of 0.832. Hu et al. [40] employed a graph adversarial transfer learning approach and achieved an accuracy of 0.835. The proposed patch-based attention framework achieves an accuracy of 0.852 on the test set used in the present study. These results provide a contextual comparison of reported performance, although differences in preprocessing, data partitioning, and other experimental settings should be considered when interpreting the numerical results.

A key characteristic of the proposed framework is its explicit patch-based representation of high-resolution fundus images. Rather than processing the entire image as a single globally resized input, the proposed framework partitions the image into non-overlapping patches and extracts feature representations using a shared ResNet-18 encoder. An attention-based aggregation mechanism subsequently assigns different weights to the patch-level representations before generating the final image-level prediction. This design enables the model to preserve localized visual information while allowing diagnostically informative regions to contribute more strongly to the final classification. The effect of this representation is further supported by the patch-number analysis presented in Section 4.4, where the 25-patch configuration achieved an accuracy of 0.852 compared with 0.817 for the whole-image (*P* = 1) baseline.

It is also important to note that several other studies have reported higher accuracy values [42,43,44,45]. These studies were not included in the quantitative comparison because their experimental settings differed from those considered in the present study. In particular, differences were observed in aspects such as the formulation of the classification task, data partitioning strategy, and class distribution. Some studies addressed binary No DR/DR classification rather than five-class severity grading, while others employed different dataset partitioning or class-balancing procedures such as data augmentation. Such differences concern the experimental setup rather than the underlying model architecture and make it difficult to interpret the reported accuracy values within the same five-class comparison. Therefore, these studies were excluded from Table 6, while the studies included in the table were selected based on their relevance to the five-class severity-grading task and the availability of a directly comparable accuracy metric.

### 4.8. Limitations

Several limitations should be considered when interpreting the results of the present study. First, the APTOS 2019 dataset contains a relatively limited number of fundus images, particularly for the Severe and Proliferative DR classes. The resulting class imbalance may affect the model’s ability to learn representative features for less frequent severity levels and contributes to the lower recall observed for these classes. In the test set, Severe DR achieves a recall of 0.294, with 12 of 17 cases classified as Moderate DR, while Proliferative DR achieves a recall of 0.394. These results indicate that, although the model achieves an overall five-class accuracy of 0.852, reliable discrimination of advanced DR severity levels remains challenging. The relatively high overall accuracy is also influenced by the strong performance on the more represented No DR and Moderate DR classes. Therefore, overall accuracy alone does not fully characterize the model’s ability to distinguish all five severity levels.

These classification errors have potential clinical implications because underestimating DR severity may affect referral prioritization and timely ophthalmic assessment. In particular, 20 of the 33 Proliferative DR cases are classified as lower severity categories, further highlighting the difficulty of identifying advanced disease. The binary DR/No-DR accuracy of 0.986 should not be interpreted as evidence of reliable five-class severity grading. Binary classification assesses whether DR is present, whereas five-class grading requires accurate discrimination among disease stages. Consequently, the high binary accuracy does not eliminate the risk of clinically significant undergrading, and the current results do not establish the suitability of the framework for clinical referral or treatment decisions.

Second, the statistical reliability of the observed performance differences requires further investigation. The ablation study in Table 5 shows an accuracy improvement from 0.836 for whole-image processing to 0.852 for the proposed attention-based configuration, while the difference between max pooling and attention-based aggregation is 0.019. Although these results favor the proposed configuration on the evaluated test set, the observed differences alone do not establish statistical significance or demonstrate that the improvements would remain consistent across different experimental settings. Therefore, the results should be interpreted cautiously, particularly given the limited size of the test set. Future work should incorporate repeated experiments, paired statistical comparisons, and evaluation on larger test datasets to provide a more robust assessment of the reliability and consistency of the observed improvements.

Third, the performance of the proposed framework depends on the selected patch configuration. As demonstrated in the patch-number analysis, different numbers of patches produce noticeably different classification performance, with 25 patches achieving the highest accuracy among the evaluated configurations. Although this analysis provides an empirical basis for comparing the evaluated configurations, the optimal number of patches may depend on dataset characteristics, image resolution, and the distribution of pathological patterns. Therefore, the selected configuration may not necessarily be optimal for other datasets or imaging settings.

Finally, the attention visualizations provide qualitative insight into the regions emphasized by the model but do not constitute direct clinical validation of lesion localization. Further investigation using lesion-level annotations or expert assessment could provide stronger evidence regarding the clinical relevance of the attended regions. Future work should therefore consider larger and more diverse datasets, multi-dataset evaluation, adaptive or multi-scale patch configurations, and approaches specifically designed to improve discrimination between closely related and advanced DR severity levels. Further experiments should also quantify the statistical reliability of performance differences and evaluate the computational efficiency of the proposed framework under standardized inference conditions.

## 5. Conclusions

Experimental evaluation on the APTOS 2019 dataset demonstrated an overall multi-class classification accuracy of 0.852 and a binary DR/No-DR accuracy of 0.986. The analysis of different patch configurations showed that the number of patches substantially affects performance, with 25 patches providing the best overall performance. Compared with the whole-image (*P* = 1) baseline, this setting improved accuracy from 0.817 to 0.852. The additional ablation study further showed that attention-based aggregation achieved the highest performance among the evaluated whole-image and patch-based configurations, with an accuracy of 0.852 and an F1-score of 0.651. The patch encoder analysis further demonstrated that performance varies across encoder architectures, with ResNet-18 achieving the highest macro-averaged Precision, Recall, and F1-score, as well as the highest overall accuracy, among the evaluated encoders. Class-wise analysis showed particularly strong performance for No DR and Moderate DR, whereas Mild, Severe, and Proliferative DR remained more challenging, particularly for the advanced severity levels, indicating that further investigation using larger and more diverse datasets is needed to improve fine-grained severity discrimination. Attention visualizations further provided qualitative insight into the model’s predictions, with correctly classified samples generally showing more focused attention on relevant retinal regions.

Future work will investigate multi-scale patch representations, lesion-aware supervision, and more advanced feature extraction and attention mechanisms to improve discrimination between closely related DR severity levels. Evaluation on additional datasets and under different imaging conditions will also be important for assessing the generalization capability of the proposed framework. Overall, the results indicate that combining localized patch-based feature learning with attention-guided aggregation is a promising approach for automated and interpretable diabetic retinopathy severity grading.

## Figures and Tables

**Table 1:** Class distribution of diabetic retinopathy severity levels in the APTOS 2019 dataset across the training, validation, and test subsets.

Diagnostic Classes	Subsets		
	Training	Validation	Test
No DR	1434	172	199
Mild	300	40	30
Moderate	808	104	87
Severe	154	22	17
Proliferative	234	28	33
Total	2930	366	366

**Table 2:** Class-wise precision, recall, and F1-score of the proposed patch-based attention model on the test set.

Diagnostic Class	Precision	Recall	F1-Score
No DR	0.980	0.995	0.988
Mild	0.778	0.467	0.583
Moderate	0.689	0.943	0.796
Severe	0.357	0.294	0.323
Proliferative	1.000	0.394	0.565

**Table 3:** Effect of the number of patches on classification performance, effective input resolution, and inference latency. Patch size is fixed at 224 × 224 pixels. Precision, recall, and F1-score are macro-averaged. Inference latency is reported as mean ± standard deviation in milliseconds per image.

Number of Patches (*P*)	Patch Size	Effective Resolution	Inference Latency (ms/Image)	Validation Accuracy	Precision	Recall	F1-Score	Accuracy
1	224 × 224	224 × 224	1.272 ± 0.015	0.822	0.646	0.583	0.604	0.817
4	224 × 224	448 × 448	2.743 ± 0.007	0.839	0.681	0.617	0.635	0.828
9	224 × 224	672 × 672	3.634 ± 0.027	0.836	0.687	0.615	0.639	0.831
16	224 × 224	896 × 896	4.841 ± 0.05	0.833	0.640	0.599	0.611	0.833
25	224 × 224	1120 × 1120	6.700 ± 0.049	0.858	0.761	0.618	0.651	0.852
36	224 × 224	1344 × 1344	8.265 ± 0.056	0.814	0.691	0.553	0.564	0.822

**Table 4:** Effect of the patch encoder architecture on classification performance using *P* = 25 patches. Overall precision, recall, and F1-score are reported as macro-averages.

Patch Encoder	Parameters (M)	Precision	Recall	F1-Score	Accuracy
ResNet-18	11.31	0.761	0.618	0.651	0.852
ResNet-50	25.67	0.651	0.617	0.622	0.822
EfficientNet-B0	4.87	0.669	0.614	0.627	0.844
MobileNetV2	3.08	0.715	0.578	0.602	0.822

**Table 5:** Effect of the patch aggregation strategy on classification performance. For the patch-based configurations, 25 non-overlapping patches of 224 × 224 pixels are used. Precision, Recall, and F1-score are reported as macro-averages.

Input	Aggregation	Precision	Recall	F1-Score	Accuracy
Whole image (1120 × 1120)	None	0.801	0.584	0.607	0.836
Patches (25 × 224 × 224)	Mean Pooling	0.668	0.609	0.619	0.825
Patches (25 × 224 × 224)	Max Pooling	0.714	0.608	0.639	0.833
Patches (25 × 224 × 224)	Attention	0.761	0.618	0.651	0.852

**Table 6:** Comparison of the proposed patch-based attention framework with previous methods for diabetic retinopathy severity grading on APTOS 2019 dataset.

Study	Method	Accuracy
Bodapati et al. [36]	Composite Gated Attention CNN	0.825
Farag et al. [37]	DenseNet-169	0.820
Hannan et al. [38]	Attention + DenseNet-169	0.832
Mustafa et al. [39]	DenseNet-121	0.725
Hu et al. [40]	Graph Adversarial Transfer Learning	0.835
Amir and Almiahi [41]	EfficientNetB3	0.840
Dixit and Jha [29]	EfficientNetB3 with squeeze-and-excitation block	0.884
Proposed method	Patch-based CNN-Attention Model	0.852

## Data Availability

The original data presented in the study are openly available at https://www.kaggle.com/competitions/aptos2019-blindness-detection (accessed on 14 September 2026).
